# The Spiny Mouse—A Menstruating Rodent to Build a Bridge From Bench to Bedside

**DOI:** 10.3389/frph.2021.784578

**Published:** 2021-11-26

**Authors:** Nadia Bellofiore, Jarrod McKenna, Stacey Ellery, Peter Temple-Smith

**Affiliations:** ^1^The Ritchie Centre, Hudson Institute of Medical Research, Clayton, VIC, Australia; ^2^Department of Obstetrics and Gynaecology, Monash University, Clayton, VIC, Australia

**Keywords:** menstruating mouse model, DHEA, abnormal uterine bleeding, preeclampsia, angiogenesis, uterus

## Abstract

Menstruation, the cyclical breakdown of the uterine lining, is arguably one of evolution's most mysterious reproductive strategies. The complexity and rarity of menstruation within the animal kingdom is undoubtedly a leading contributor to our current lack of understanding about menstrual function and disorders. In particular, the molecular and environmental mechanisms that drive menstrual and fertility dysregulation remain ambiguous, owing to the restricted opportunities to study menstruation and model menstrual disorders in species outside the primates. The recent discovery of naturally occurring menstruation in the Egyptian spiny mouse (*Acomys cahirinus*) offers a new laboratory model with significant benefits for prospective research in women's health. This review summarises current knowledge of spiny mouse menstruation, with an emphasis on spiral artery formation, inflammation and endocrinology. We offer a new perspective on cycle variation in menstrual bleeding between individual animals, and propose that this is indicative of fertility success. We discuss how we can harness our knowledge of the unique physiology of the spiny mouse to better understand vascular remodelling and its implications for successful implantation, placentation, and foetal development. Our research suggests that the spiny mouse has the potential as a translational research model to bridge the gap between bench to bedside and provide improved reproductive health outcomes for women.

## Introduction

The menstrual cycle is a process in which the uterine lining sheds, regenerates, and terminally differentiates in preparation for pregnancy. Unsuccessful pregnancy results in necrosis and shedding of the functional layer of the uterus, the endometrium. This culminates in cyclical uterine bleeding termed menstruation. Menstruation occurs almost exclusively in higher order primates, including humans and Old-World monkeys, with several species of bat and the elephant shrew being the few exceptions (1–5).

As unique as our non-pregnant cycles are, so too are our pregnancies with menstrual species presenting some of the most complex and metabolically demanding gestational processes in mammals (6–9). Consequently, the absence of menstruation as the preferred female reproductive strategy in mammals has restricted the advancement of our knowledge and understanding of the biology of menstruation, and especially the targeted development of therapeutics for menstrual and associated pregnancy disorders. While the need for a more thorough understanding of women's health is clear, the dearth of appropriate non-human laboratory models has slowed the progression of menstrual research.

The recent discovery that a small rodent, the common spiny mouse (*Acomys cahirinus*), has a naturally occurring menstrual cycle provides a rare opportunity to use a small laboratory animal in studies of female reproductive biology (10). An in-depth characterisation of this singular phenomenon reveals similarities in both physiological and behavioural aspects of menstruation with higher order primates including humans (11, 12). Further, certain likenesses to primate pregnancy (13, 14) places the spiny mouse as a promising candidate for examining various aspects of women's reproductive health. This review will discuss the advantages of using this now underutilised laboratory rodent in menstrual and gestational research, with particular emphasis on correlating the unexplored linkages between menstrual health and pregnancy outcomes. We will examine how the unique endocrinology of this species may also be leveraged for studying reproductive and inflammatory disorders to highlight to the broader scientific community the untapped potential of this rodent for biomedical research.

## Disrupted Cycles, Disrupted Lives

In the collaborative Global Burden of Diseases Project (15, 16), almost 500 disabling diseases and injuries were subjected to a comprehensive statistical analysis of prevalence and mortality, including disability-adjusted life years (DALYs). Menstrual disorders, including Abnormal Uterine Bleeding (AUB) were not classified among them. AUB is defined as a change in the frequency, duration and/or amount of blood loss in menstruation. and can be further classified according to the International Federation of Gynaecology and Obstetrics in 2011 PALM-COEIN system (Polyp, Adenomyosis, Leiomyoma, Malignancy and hyperplasia, Coagulopathy, Ovulatory Dysfunction, Endometrial, Iatrogenic, Not yet classified) (17). Under these categories, up to 80% of menstrual bleeding disorders can be accounted for (17). Many ovulatory disorders can be attributed to aetiologies such as polycystic ovarian syndrome, extreme weight fluctuations, stress or other endocrinopathies to result in disrupted cycles. A spectrum of deviations from the “normal” menstrual period manifests in patients from extremely light or infrequent bleeding (amenorrhea) to heavy menstrual bleeding (HMB, replacing previously used menorrhagia). HMB is often the simplest aspect of AUB to assess the burden of disease as it generally has the greatest impact on daily function and quality of life (17).

A systematic review analysed available data from 1980 to 2005 (18) to address a disturbing gap in awareness in the public, medical and political sectors provides conservative estimates of the global burden and Health-related Quality of Life (HRQoL) of AUB. Prevalence of AUB was estimated at 10–30% of patients in regions of Europe and the United States. Unsurprisingly, women who sat below the 25th percentile for HRQoL were negatively impacted in their work productivity. The authors estimated the annual direct financial costs (such as visits to medical practitioners, surgeries and medical interventions) of $1–1.55 billion. Indirect costs (such as workplace absenteeism) could be as high as $12–36 billion, with AUB patients reported to work 3.6 weeks less per year than age-matched women without AUB. Furthermore, hysterectomy or endometrial ablation remain the prominent non-medicinal treatments for AUB, with almost 90% of women hospitalised with AUB undergoing surgery, including hysterectomy, which is the second most performed gynaecological procedure in the US. These values clearly reflect not only the dire need for further research into alternative methods of treatment for AUB, but a shift in global perception of the degree of debilitation caused by menstrual cycle dysregulation. To achieve this, a comprehensive understanding of menstruation across a physiological, cellular, and biomolecular level is critical, though are not easily studied among women. For this research, especially those which inform new treatments, appropriate animal models are required.

## The Menstrual Cycle in The Animal Kingdom

To understand how we can best examine disorders of menstruation and pregnancy with our current available resources, we first need better to understand the intricacies of the menstrual cycle. The menstrual cycle involves complex hormonal interactions between hypothalamus, pituitary gland, ovaries, and uterus. A comprehensive description can be found in Johnson (19). Briefly, the major uterine morphological and physiological changes governed by ovarian steroids oestradiol-17B (E_2_) and progesterone (P_4_). The menstrual cycle comprises both uterine and ovarian distinctive phases under direct stimulation from pituitary hormones. The beginning of a new fertile cycle in all menstruating species is marked by shedding of the superficial layer of the endometrium in the uterine cycle; i.e., menses or menstruation. Menses corresponds with the follicular phase of the ovarian cycle, where follicle-stimulating hormone (FSH) secreted from the anterior pituitary causes a gradual increase in follicular recruitment and growth. As follicles mature, they in turn secrete increasing levels of E_2_, resulting in a rapid thickening of the endometrial stroma during the corresponding proliferative phase of the uterine cycle. High E_2_ initiates a surge of luteinising hormone (LH) from the anterior pituitary gland, triggering the release of the oocyte mid-cycle during the ovulatory phase. The remnant of the ovulatory follicle forms a functional corpus luteum, which secrets both sex steroids, with P_4_ the dominant hormone of the luteal phase in the ovarian cycle (19). It is this process that delineates menstrual species from most other mammals (20).

Viable placental and foetal development rely on the functional transformation of the endometrium under the influence of P_4_ from the corpus luteum. The current leading theory suggests that menses is the by-product of spontaneous decidualisation of the endometrium. During the secretory/luteal phase of the uterine/ovarian cycle (respectively), endometrial stromal cells undergo spontaneous decidualisation, a terminal metamorphosis to facilitate embryo implantation (21–24), which is a rare and pre-emptive process occurring only in menstruating species. This terminal differentiation of the endometrial stroma allows cells to form a hospitable niche for the implanting embryo, while also selecting out those of poor quality and reducing the risk of non-viable pregnancies (22, 25). Simultaneously, endocrine signalling promotes substantial angiogenesis; the creation of new blood vessels from the uterine artery and results in the formation of spiral arteries (26). The unison of these cyclical processes is crucial in preparing the endometrium for a successful pregnancy.

Concurrently, uterine angiogenesis during the menstrual cycle culminates in the development of large, visibly coiled spiral arteries. Further substantial vascular changes during early gestation, known as spiral artery remodelling, are essential to support adequate gas and nutrient exchange with the growing foetus. Without a human chorionic gonadotrophin (hCG) rescue signal secreted from an implanted blastocyst, the corpus luteum degenerates, and P_4_ is rapidly withdrawn. This withdrawal causes necrosis of endometrial decidual cells, and the degradation of the stromal matrix in an inflammatory event similar to the tissue repair mechanism seen in wound healing. As the supporting uterine architecture is broken down, so too are the newly formed blood vessels, resulting in the flow of blood that we observe during menses. A new fertile cycle is now initiated.

Of nearly six thousand identified mammals in the world, <2% have adopted the reproductive strategy of cyclical shedding and rebuilding of the endometrium (10). Almost all identified menstruating species belong to the primate order. The handful of exceptions, which includes a few species of bats and, perhaps, the elephant shrew (1, 3–5, 27), do little to help resolve the evolutionary enigma of menstruation. While most have common traits suggesting the existence of a phylogenetic link, a “one-size-fits-all” explanation for why some species adopted this strategy has yet to be elucidated. An in-depth comparison of biological commonalities is reviewed in Bellofiore et al. (20) from which we can infer that while spontaneous decidualisation, mode of placentation and offspring maturity at birth are often comparable in menstruating mammals, there are a number of grey areas. For example, all menstruating species identified to date appear to have haemochorial placentation, but not all species with haemochorial placentation menstruate, for example mice and guinea pigs. This intent to find common ground among menstruating species is further obscured by the spiny mouse.

## Menstruating Mice

Rodents do not naturally menstruate, the only exception to date being the spiny mouse (10). Currently, there are few established captive colonies of spiny mice across the world, with various species used to study different aspects of biology. The species described in this review, unless otherwise specified, refers to that of an in-house derived research colony of common spiny mice at Monash Medical Centre, Melbourne, Australia. This colony was established in 2001 from 5 breeding pairs, and since then have not had new genetic material added. The implications of this potential genetic bottle-necking have been previously discussed elsewhere (28), as has a comparison of husbandry with that of other known colonies (20, 28).

The common or Egyptian spiny mouse (*Acomys cahirinus*) is a ground-dwelling rodent native to regions of Africa and the Middle East. It is one of over 20 species in the genus *Acomys*, with names often reflecting geographical dispersion or coat colours (29). The species' name is derived from their dorsal exterior coat; thick, rigid, and spine-like hairs displacing the soft neonatal fur at ~30–60 days. The spiny mouse is still a relative novelty in scientific research and possesses many remarkable traits for a rodent. Spiny mice were initially used to study diabetes mellitus due to their tendency for obesity and pancreatic hyperplasia when fed a high sugar diet (30, 31). More recently, spiny mice have been discovered to have skin autotomy, demonstrating scar-free wound healing for the first time in a mammalian species (32).

**Figure 1:**
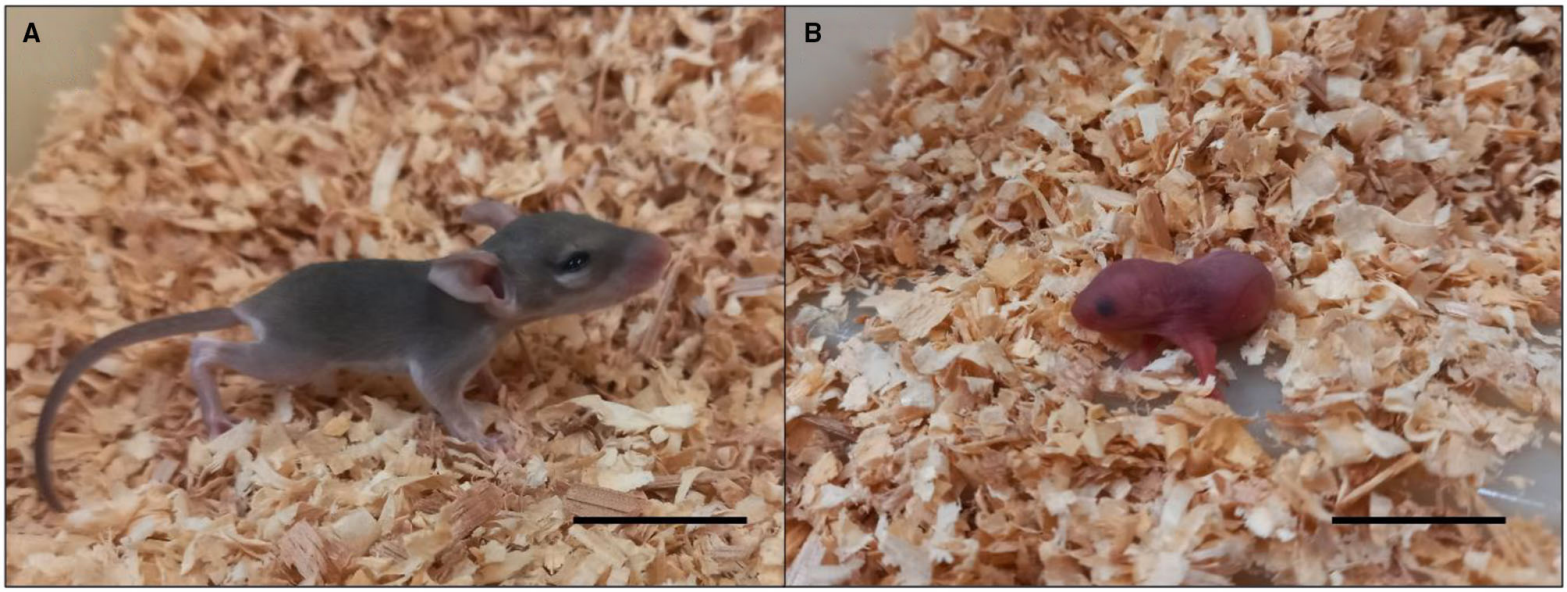
Spiny mouse **(A)** and mouse **(B)** pups on the day of birth. Spiny mice are covered in fur, eyes and ears opened, mobile with limbs fully developed and functional, weighing ~5–6 g in litter sizes of 1–5. The mouse pup has not developed fur, or mobility, with eyes and ears closed. Mice weigh ~1–2 g in litter sizes of 4–12. Scale bars = 1 cm.

Their distinctiveness extends further to their reproduction. Dams have a relatively long gestational period (38–39 days), approximately twice that of a standard laboratory mouse (*Mus musculus*), and deliver on average 2–3 developmentally mature (precocial) pups per litter (33). In stark contrast to conventional rodents, spiny mouse pups are born in a similar advanced state of development to human babies, having completed organ development *in utero*, being completely mobile, and with eyes and ears open (Figure 1). The lower numbers of offspring in spiny mouse pregnancy likely reflects a greater placental investment, including increased ratio of labyrinth to spongy zone regions (13), and their surprising capacity to synthesise adrenal hormones cortisol and dehydroepiandrostenedione (DHEA) (34, 35), which result in *in utero* maturation of most organs, including lungs, kidneys and ovaries prior to birth (36–38). The precocious nature of the spiny mouse is preferred to the laboratory mouse to study foetal development. We have used this rodent to establish models of perinatal injury, including intrauterine growth restriction and birth asphyxia (39, 40).

The recent observation of cyclical spontaneous decidualisation and ensuing menstrual bleeding in spiny mice (10) confirmed *A. cahirinus* as the only rodent with a naturally-occurring menstrual cycle. In other rodents, decidualisation does not occur until embryo implantation. However, artificial decidualisation and menstruation can be experimentally induced in *M. musculus*, as fist described by Finn and Pope (41). Briefly, a decidual reaction in ovariectomised mice was initiated using E_2_ and P_4_ injections, followed by physical stimulation of the endometrial stroma via arachis oil injection into the uterine lumen. When hormonal support was withdrawn, the decidualised cells were no longer supported, resulting in menstrual-like shedding. This model has been further refined using E_2_ priming prior to insertion of slow-release progesterone implants, allowing for a more rapid hormonal withdrawal and greater physiological relevance to true menstruation (42). Further alterations to have been made to the mouse model of menstruation which have contributed to a greater understanding of the role of hormones. One study used in-tact females and induction of pseudopregnancy to elevate endogenous P_4_ and demonstrate menstrual shedding following a natural declination (43), while others have generated data supporting that P_4_ support is time-sensitive, and shedding becomes irreversible after a time-critical window (44). Variations of the mouse model of menstruation have since been used to establish new models of endometriosis through intraperitoneal injection of menstrual debris from experimentally induced donor mice (45). Importantly, these models have made substantial contributions in our understanding of androgens in endometrial repair (46), neutrophil and macrophage promotion of angiogenesis during endometriotic lesion development through cytokine secretion (47) and altered expression profiles for genes regulating the immune system, cell adhesions, proliferation and angiogenesis in the endometriosis phenotype (48). Undoubtedly, these induced models have led to vital insights into the menstrual regulation and pathologies of menstrual disorders. However, artificial models have their limitations, especially regarding menstruation as a lifetime recurrence, the relevance of induced decidualisation and use of ovariectomised models. These limitations have been extensively discussed elsewhere (11, 20, 28), as have the advantages of a using naturally menstruating rodent, the spiny mouse.

Relative to other menstruating species, the spiny mouse menstrual cycle is brief, lasting on average 9 days and ranging from 6 to 12 days in healthy, sexually mature females. The morphology and function of the decidual cells in the spiny mouse closely mimics decidualisation in primates; it is stimulated by a significant rise in progesterone, and recognised by secretion of biomarkers prolactin and interleukin-11 (10, 11). Comprehensive histological and morphological analysis of the spiny mouse uterine architecture across the menstrual cycle confirmed similarities to primate menstruation in the breakdown, repair, and rebuilding of the endometrium. The spiny mouse demonstrates focal “piecemeal” shedding in progressive waves along the uterus, with peak inflammatory influx of neutrophils occurring mid-menstruation and simultaneous repair occurring in the epithelium (11). Similarities were also evident in the uterine secretion of inflammatory and repair markers interleukin-8 and macrophage inhibitory factors, as well as the localised focal shedding and adjacent repair of the endometrium (11). These studies also demonstrated behavioural and physiological similarities between spiny mice and other naturally menstruating species, in that changes in food consumption, weight fluctuations, anxiety, and exploration are driven by menstrual cycle stage and suggestive of a human-like premenstrual syndrome (PMS) (12). Having a non-primate model of PMS in a species with a short menstrual cycle allows an unexpected opportunity for a comprehensive examination of the influences of molecular and endocrinological changes during the menstrual cycle on the behavioural repertoire of individuals during the transition from sexual maturation into adulthood. Such potential warrants further investigation and is discussed more thoroughly by Bellofiore et al. (12, 20).

## An Emerging Model of Abnormal Uterine Bleeding

Studies using the spiny mouse have identified the natural formation of spiral arteries prior to menstrual onset for the first time in a rodent (11, 20). A combination of histological techniques, including immunofluorescent double-labelling for alpha-Smooth muscle actin and vascular endothelial growth factor, has provided evidence of cyclical vascular remodelling concurrent with spontaneous decidualisation occurring naturally in the spiny mouse. This strongly encourages the use of the spiny mouse as a model for uterine vascular studies.

Individual spiny mice have notable variation in menstrual blood loss (11). Within the well-characterised Monash colony, menses is best identified under light microscopic analysis of haematoxylin and eosin-stained vaginal lavages, with frank menstrual blood observed at the vulva a rarer occurrence. In fact, the level of individual bleeding between spiny mice is remarkably varied, with up to 10% of females exhibiting what is most appropriately describe as heavy menstrual bleeding. In these females, menstrual blood can be viewed either at the vagina or in the lavage sample itself, for prolonged periods of time. The reverse is also true, in that a similar proportion of females demonstrate light menstrual bleeding whereby relatively few erythrocytes can be identified in lavage samples or a menstrual bleed that lasts less than a day. Differences in menstrual blood loss between individuals is also observed in women (49). A small animal model of natural AUB, not an experimentally induced AUB, may provide interesting new insight into the genetic or environmental origins of such deviations from the norm, or even help us to better understand why these variations exist at all.

The evolution of a wide spectrum of menstrual blood loss, which exists among women, has yet to be definitively explained, as is the variation in the timing, onset, and heaviness of an individual's menstrual period when an underlying pathological cause is not identified (17). It has been argued that menstrual bleeding has evolved not necessarily as a selective advantage in itself, but rather as a by-product of another biological process (spontaneous decidualisation); it has merely evolved because it does not provide a disadvantage to survival (23). However, logic would then suggest that a small rodent species, likely to be the target of numerous ground and aerial predators, would find it a significant disadvantage to leave a trail of blood each menstrual cycle, providing both visual and olfactory signals. We must then assume that the risk of predation is outweighed by the benefit of spontaneous decidualisation and spiral artery formation prior to conception. The question remains: what are these benefits? Having observed such a large natural variation of bleeding in a species which has not been subjected to selective pressures of environment and predation, we hypothesise that females with heavy menstrual bleeding have a selective advantage for breeding. Their vessels undergo more extensive remodelling, resulting in optimal placental nutrient and gas exchange and, hence, larger and/or more offspring can be supported.: The subsequent excessive blood loss during menstruation stems from increased spiral artery formation and a thicker endometrial lining, both of which are stimulated by the androgen DHEA.

## DHEA: Making Menstruators and Augmenting Angiogenesis

### DHEA and Reproduction

Current knowledge emphasises the roles of sex steroids oestrogen and progesterone in female fertility, but largely ignores the importance of DHEA. DHEA and its metabolite for storage in the bloodstream, DHEA-Sulphate (DHEA-S), are among the most abundant circulating steroids in humans (50). A ubiquitous androgen primarily derived from the adrenal glands, and perhaps by the gonads, DHEA production is significantly increased during the early stages of sexual maturation and is an important precursor in the synthesis of sex steroid hormone testosterone and oestrogen (51).

DHEA is one of the main androgens elevated during adrenarche. This is a process of sexual maturation related to puberty during which the production of androgens is increased, and is typically specific to higher order primates (52). The association between adrenal maturation, time to menarche (the first period), and female fertility, while undoubtedly intrinsically linked, has not been well-examined in menstruating species. While Old World monkeys (humans included) have a distinctive prepubertal adrenarche, New World monkeys and other non-menstruating species lack enzymes in the steroidogenic pathway which reduce the ability of the adrenal glands to produce DHEA. Previous studies have confirmed that the spiny mouse has P450c17 activity and produces DHEA *in utero* (35). In contrast, non-menstruating marmosets have significantly reduced circulating DHEA due to a deficiency in the key enzyme P450c17 (53).

A further commonality between menstruating species is the production of 1–2 offspring per pregnancy. This is a repeated theme in the menstruating spiny mouse, littering on average 2–3 pups (though this can range from 1 to 5). Conventional laboratory rodents which lack the capacity to synthesise DHEA (34, 36) do not spontaneously decidualise or menstruate, and have litters ranging from 8 (mouse) to 18 (Rats). This demarcation between species suggests DHEA has a central role in the menstrual cycle [reviewed in detail in (28)] and has an important advantage for conception and maintenance of precocial foetal development.

Decidualisation of the uterine stroma during the menstrual cycle in preparation to support pregnancy relies in part on the actions of androgens. In a recent *in vitro* study, androgens were shown to play a pivotal role in enhancing decidualisation through intracrine action (54), as well as their role in regulating repair mechanisms during menstruation (46). Capable of acting as an oestrogen receptor agonist, DHEA is thought to be a key substrate for oestrogen biosynthesis in postmenopausal women (55, 56). Interestingly, through oestrogenic conversion, it also displays immunomodulatory properties, dampening excessive inflammatory responses in mouse models of impaired wound healing (57). The importance of DHEA has also been previously highlighted in regards to the central nervous system and neurodevelopment of the foetus, as well as for placental biosynthesis of oestrogens (58).

### DHEA: An Angiogenic Androgen

Links between adrenal regulation of the uterine vascular network and the corresponding degree of menstrual bleeding have not been identified. However, previous studies have shown that DHEA promotes angiogenesis in many species and tissue types (59, 60). The stimulatory actions of DHEA on uterine vessel growth have yet to be extensively examined, as methods for visualising the feto-placental unit vasculature during pregnancy in naturally menstruating species have not been possible. Furthermore, not without some merit, DHEA has been labelled a “human hormone”; distinct in its synthesis and circulating levels from other mammals. This makes it extremely challenging to study this natural hormonal interaction in less immediate target tissues, such as the uterus, to follow long-term biological processes, such as puberty and pregnancy. Thus, our understanding of how altered androgenic input may impact vascularity and what the implications are for foetal development has been significantly slowed.

DHEA promotes angiogenesis. It was recently identified to increase endothelial proliferation by 30% in bovine aortic vascular endothelial cells *in vitro*, as well as promoting the growth of new primary capillaries (60). The investigators also demonstrated that DHEA enhances angiogenesis by measure of increased vessel density in an *in vivo* chick chorioallantoic membrane assay. DHEA has also been implicated in stimulation of vascular endothelial growth factor (VEGF), a potent angiogenic protein in the human menstrual cycle (26) and identified in the endometrium and spiral arteries in the spiny mouse (11). In a study of natural killer cells obtained from healthy and Alzheimer's patients, incubation with DHEA-S increased VEGF production in a dose-dependent manner (59). This is of particular importance, as VEGF deficient mice are incapable of developing the appropriate vascular network needed to support pregnancy and subsequently abort (26). VEGF production and vascular permeability in the endometrial stroma is also increased in response to oestradiol (26). DHEA has been shown to act through both the oestrogen receptor and as a substrate for oestrogen synthesis (56). This is suggested in androgen receptor knockout (AR^−/−^) mice, whereby AR^−/−^ females were still able to conceive, but produced less pups and had significantly reduced uterine area (including the endometrium). This may be due to DHEA only exhibiting actions as the oestrogen receptor (61). Furthermore, endometrial hypertrophy due to oestrogenic overstimulation has not been associated with DHEA treatments (62). Thus, the discovery of the role of DHEA as a potential regulator of the uterine microenvironment presents an exciting research opportunity. This is particularly pertinent in the menstruating spiny mouse, given they retain enzyme P450c17 for DHEA synthesis.

### Developing Preeclampsia

Preeclampsia (PE) is a severe disorder affecting 1 in 20 pregnancies (63), and characterised by dangerously high maternal blood pressure, organ failure, and foetal compromise. Complications from PE is a crisis spanning even developed countries; it accounts for 20% of maternal deaths in the United States and 60,000 maternal deaths annually (64–66). As the primary intervention is delivery of the placenta, PE is also the largest cause of preterm birth, inflicting adverse neurological outcomes and respiratory disorders upon already vulnerable babies (67). Therefore, PE poses an imminent threat to the immediate and long-term health of many mothers and babies.

The foundations for PE are laid prior to pregnancy, with poor functional transformation of the endometrium in preceding menstrual cycles (68). In turn, the endocrine signalling needed to promote sufficient decidualisation, angiogenesis and spiral artery remodelling, are lost. The absence of these interactions ultimately leads to shallow invasion of the endometrium by the embryo within the first week of pregnancy, and subsequently PE (26, 68, 69).

In healthy pregnancies, extravillous trophoblasts migrate through the decidua to colonise the myometrial spiral arteries, forming intraluminal plugs before eventually replacing the maternal endothelium. During this proteolytic invasion, the elastic and muscular tissues of the spiral arteries are destroyed to incorporate the cytotrophoblasts into the vessel walls before finally reconstituting these vessels without maternal vasomotor control (70, 71). These changes facilitate a low-resistance, high volume blood flow system to meet the vascular demands of a growing foetus.

PE is a disease of heterogeneity, most commonly diagnosed between 20 weeks gestation and up to 48 h postpartum (64, 71). Superficial penetration of the cytotrophoblast results in widespread endothelial dysfunction and placental malperfusion and ischemia; phenotypes shared by both PE and intrauterine growth restriction (IUGR) pregnancies (often simultaneously) (71). Pinpointing the failure of the uteroplacental arteries to dilate has been the subject of much controversy. Zhou et al. suggested that the extravillous trophoblast of preeclamptic patients have reduced expression of adhesion molecules, including E-cadherin, platelet- and vascular-endothelial adhesion molecule-1 (PECAM-1 and VECAM-1, respectively) (72). However, Lyall et al. could not recapitulate this, finding no differences in expression in the trophoblast cells between normal and preeclampsia or IUGR patients (73). The role of nitric oxide synthesis is also in question, as uteroplacental arteries in guinea pig models dilate when stimulated by the invading trophoblast, as well as inducing preeclampsia and IUGR phenotypes in guinea pigs and rats when nitric oxide synthase was inhibited (74–76).

Furthermore, maternal factors must be considered in preeclampsia development, as chronic hypertension, diabetes, renal disease, very young or advanced age are all maternal risk factors contributing to likelihood of disease onset (64, 71). Immune maladaptation is also a theory, with Kaufman and Huppertz hypothesising that reduced trophoblast invasion of uteroplacental arteries leads to an accumulation of apoptotic interstitial trophoblast and excessive recruitment of macrophages. Macrophage activation then leads to further macrophage attraction in a positive feedback cycle, preventing further endovascular invasion.

Brosens urged consideration of the role of progesterone-dependent decidual cells in preparation for the inflammatory event of early pregnancy (77). Decidual cells, which are resistant to oxidative stress, form a cuff around the spiral arteries during the secretory phase of the menstrual cycle. This cuff then provides potential histotrophic support of the early conceptus and for local chemokine secretion to trigger an influx of specialised uterine Natural Killer (uNK) cells; immune cells that aid in vascular remodelling through secretion of growth and angiogenic factors. Brosens also advocates that PE is undoubtedly a disease of early pregnancy, and caused by the imbalance of low angiogenic factors and high antiangiogenic factors (64). Brosens suggested that inadequate blood vessel remodelling may be attributed to impaired decidualisation and uNK function. Additionally, immune imbalance is also implicated in PE pathogenesis as a result of placental ischemia (77). Uncontrolled, chronic inflammation results from a T-helper (Th) cell reversal, whereby the ratio of Th-1 to Th2 cells is high. Th-1 cells increase secretion of proinflammatory cytokines, including interleukins (IL)-6 and−17 and Tumour Necrosis Factor-alpha (TNF-α). A subsequent decreased regulatory immune cells influenced by Th-2 cell action, when combined with this, perpetuates the unchallenged production of reactive oxygen species culminating in the hallmark symptoms of PE, hypertension and endothelial dysfunction (78).

Importantly, there are hints of a relationship between endometrial and pregnancy pathologies, including an increased risk of PE based on the “lightness” of a woman's menstrual period, as these women may have inadequate remodelling of uterine blood vessels (77, 79, 80). A recent theory suggests that cyclical menstruation evolved as a means of preparing the uterus for the impending hyperinflammation and tissue ischemia associated with invasive placentation (77). This protective process, known as uterine preconditioning, would explain why the prevalence and severity of preeclampsia (PE) is highest in first pregnancies and adolescents who have had insufficient decidualisation and corresponding vascular preparation (81). In first pregnancies, multiples (i.e., carrying more than one baby), and in women conceiving through *in-vitro* fertilisation, the risk of developing PE is increased 2–3-fold. In multiple pregnancies, increased placental mass causes increased soluble fms-like tyrosine kinase-1 (sFlt-1), impairing angiogenesis (82–85).

**Figure 2:**
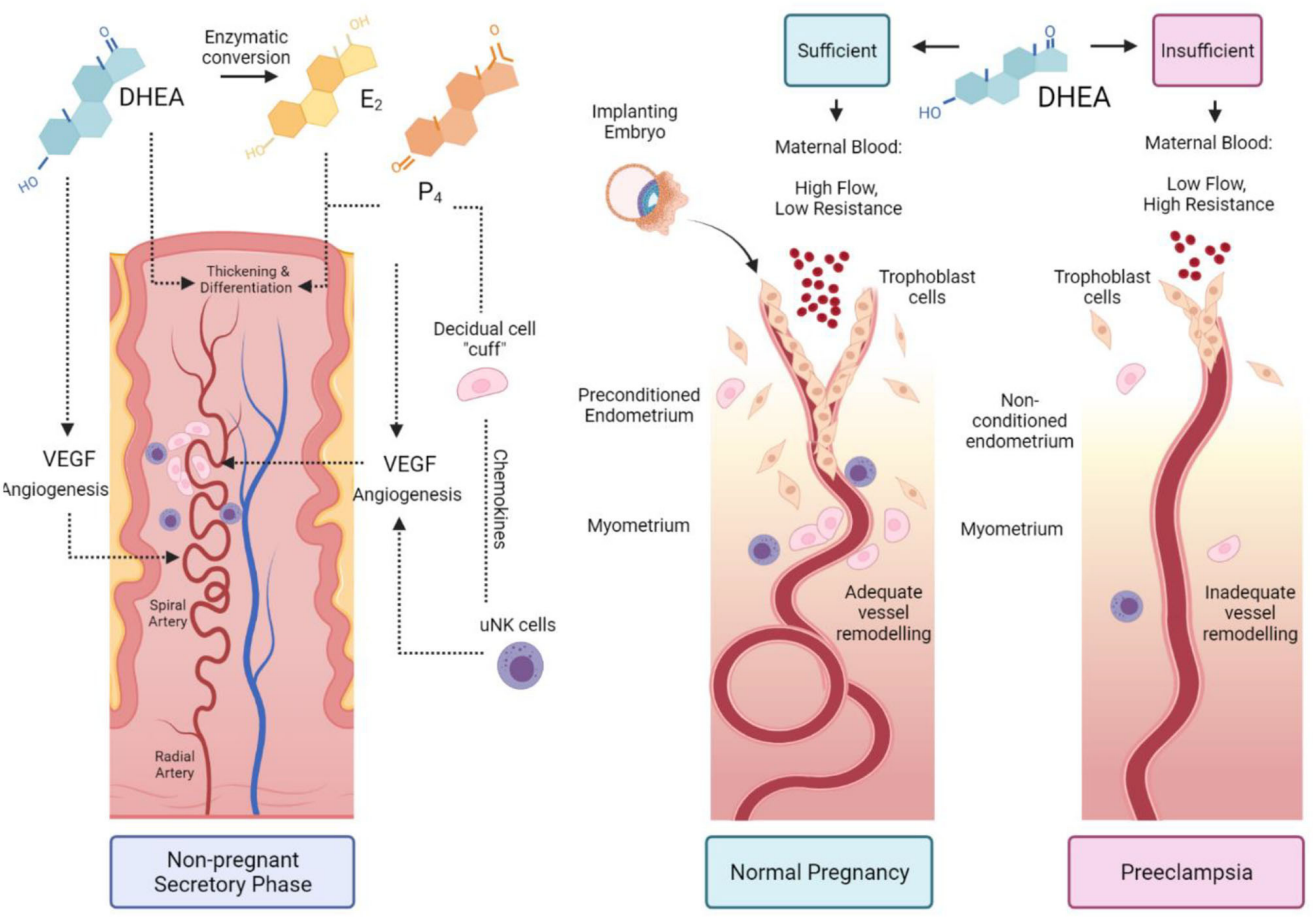
The proposed role of DHEA in preconditioning the uterus for pregnancy. During the secretory phase of a normal menstrual cycle, DHEA acts via an androgen receptor or through conversion to oestrogen and through the oestrogen receptor to stimulate angiogenesis and spiral artery remodelling. Oestradiol (E_2_) and progesterone (P_4_) promote endometrial thickening and differentiation during spontaneous decidualisation. The decidual cells form a cuff around spiral arteries and recruit specialised uterine natural killer (uNK) cells to further promote vessel remodelling (*Left-hand panel*). Sufficient DHEA primes the uterus in the preceding menstrual cycles for embryo implantation. Trophoblasts invade through the remodelled vessels up to the myometrial layer and enable high maternal blood flow with low vascular resistance (*Middle panel*). Insufficient DHEA causes poor uterine preconditioning; low DHEA and reduced conversion to E_2_ impairs decidualisation, uNK recruitment, angiogenesis and vessel remodelling. Trophoblast invasion is shallow, with high blood pressure due to vascular resistance, resulting in preeclampsia (*Right panel*).

Combining the knowledge that DHEA is a stimulant of angiogenesis and decidualisation with Brosens' preconditioning theory, we propose a novel theory: women with low DHEA have impaired preconditioning, endometrial and vascular remodelling, resulting in increased risk of preeclampsia. Our hypothesis is presented in Figure 2.

Kaufman and Huppertz astutely summarise the most prominent drawbacks in using human tissue alone to study preeclampsia. Placental samples, which are readily available after delivery, do not cover the primary zone of interest, and placental bed biopsies obtained during caesarean section are not robust representatives of the entire organ. Even for non-preeclampsia uterine studies, whole uterine samples are difficult to obtain. If hysterectomised organs are available, often control cases are limited, as patients rarely elect to have a hysterectomy in uncomplicated circumstances (71). These areas could be greatly benefitted from using an appropriate animal model in this area of research. O. Indeed, in current animal models, often only a single clinical symptom of PE (i.e., high blood pressure or renal dysfunction only) can be induced through genetic manipulations or administration of compounds. However, these models do not sufficiently mimic disease progression (86). The success of developing novel therapeutics as either prophylactic measures or treatments for PE in humans relies largely on extensive preclinical trials in appropriate whole-animal or artificial organ systems (e.g., 3D cell-culture or organoids). Here lies obvious advantages of the spiny mouse, including in the context of preeclampsia; the possibility of studying control or manipulated organs in their entirety, which may also allow the possibility of whole feto-placental unit dissection and analysis with modern imaging technology.

As menstruation and PE are almost exclusive to higher order primates (23) there have been limited advances in understanding this disease pathophysiology, particularly in being able to use the menstrual cycle to screen for PE and developing new predictive tests and treatments. There are no current medical tests to identify women at increased risk of developing PE before pregnancy. Therefore, non-invasive monitoring of the menstrual cycle and subsequent degree of bleeding presents a potentially underutilised, yet pivotal, predictor in the development of PE. PE has is not yet been confirmed in spiny mice, though idiopathic maternal death during or immediately following the birth of pups in our colony has been observed. Nonetheless, the spiny mouse may provide fundamental preclinical data on long-term menstrual physiology and associated birth outcomes, enabling identification of new biomarkers and aid in developing surveillance protocols for at-risk females.

## A Hybrid Model for Pregnancy and Implantation

The recent discovery of a naturally menstruating laboratory rodent, the spiny mouse, provides a new investigative tool to address pregnancy and implantation-related disorders. Not only has the cyclical assembly of spiral arterioles in the secretory phase of the menstrual cycle been confirmed in this species through histological assessment, but further work characterising early pregnancy reveals further primate similarities. Immunohistochemistry using alpha smooth muscle actin (aSMA) and cytokeratin enabled visualisation of vascular and epithelial structures, respectively, during the menstrual cycle (11) and pregnancy (87). The early pregnant spiny mouse (day 10 of a 38-day gestation) demonstrates an absence of aSMA staining during early placental development, whereby trophoblast invade the maternal vasculature. As in humans, the breakdown of the vascular smooth muscle surrounding spiral arteries allows for high-pressure, low-resistance placental perfusion (87). Observing this in spiny mice is indicative of potentially conserved vascular mechanisms for the development of a viable feto-placental unit, and may be used to study the resulting phenotypes from arteriole remodelling impairment and dysfunction.

Spiral arteriole remodelling is not mutually exclusive from spontaneous decidualization; the latter in fact is thought to support the unique angiogenic process. The correlation between species which exhibit a spontaneous decidual response, as opposed to those which do not, demonstrates a potential commonality between seemingly unrelated menstruating mammals. This suggests that spontaneous decidualisation evolved as a pre-emptive defence mechanism against the aggressive nature of embryonic invasion during pregnancy and the placental type of the species (23). However, recent reports show that, despite the presence of spiral arterioles and remodelling during early pregnancy, the spiny mouse has a shallow, eccentric embryo invasion of the endometrium similar to their murid relatives (87). The spiny mouse therefore neither recapitulates the primate nor the rodent reproductive strategies perfectly, but demonstrates aspects of both in terms of vascular remodelling (primate) and implantation (rodent).

**Figure 3:**
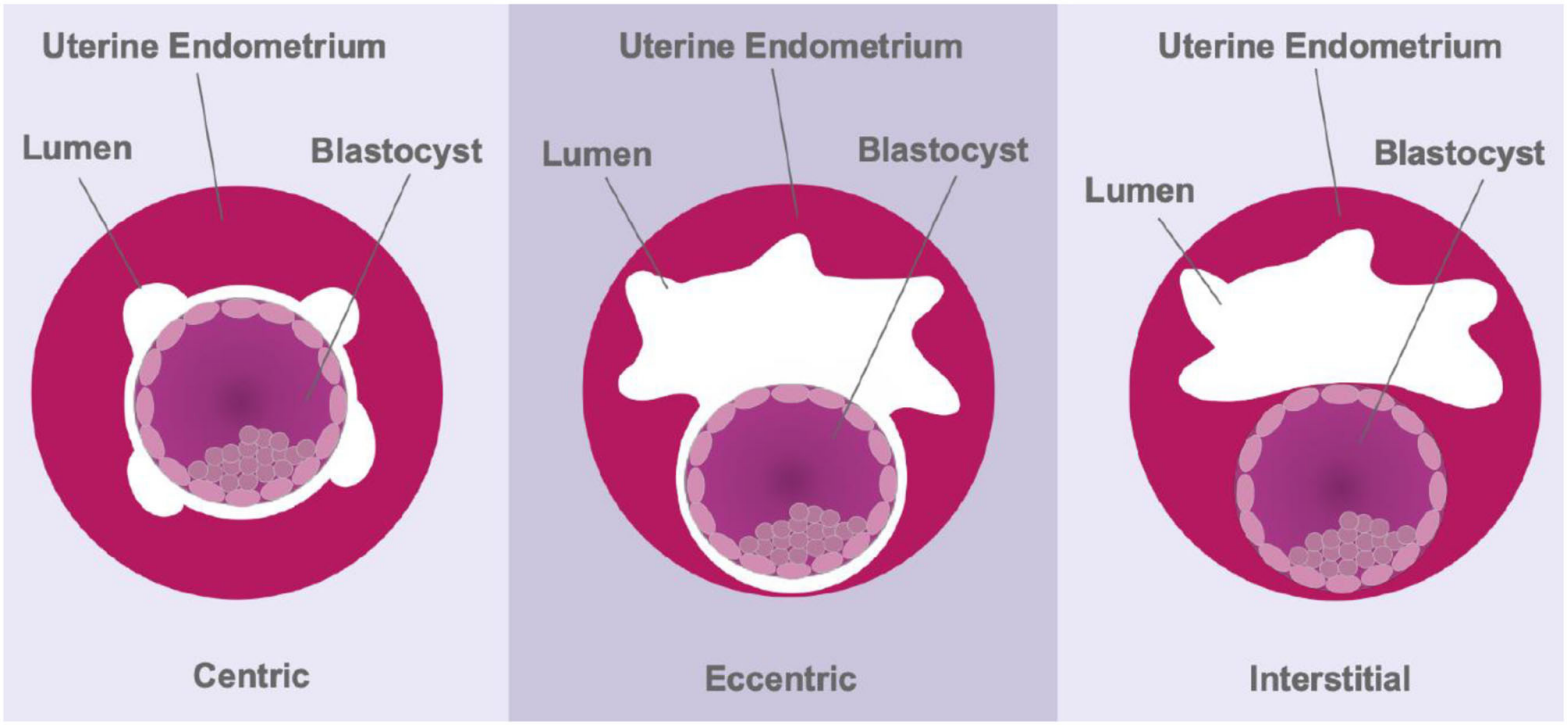
Schematic representing the three modes of implantation. During centric implantation, the embryo is superficially attached to the uterine epithelium and trophoblasts do not invade the endometrium (cows and ewes). Eccentric implantation involves the formation of a tear-drop shaped cavity within the endometrium with a moderate degree of trophoblast invasion (most rodents). During interstitial implantation, the blastocyst completely embeds itself within the endometrial stroma with trophoblasts invading to the basal endometrium (primates).

The depth of trophoblast invasion can differ dramatically between species. Menstruating primates and guinea pigs present with aggressive, interstitial embryo implantation (20, 88), whereas most rodents show eccentric implantation with moderate trophoblast invasion (Figure 3). Moreover, adhesion to, and invasion of, the uterine wall occurs within 6 h in rodents, which limits the use of this order for understanding the underlying physical mechanisms involved in early stages of human implantation (89). Cows and ewes, on the other hand, show centric embryo implantation with no invasion of the uterine epithelium. In these species the foetus grows within the uterine lumen. Non-menstruating primates such as common marmosets have been used as *in vitro* models of primate embryo adhesion and protein secretion (90). Marmosets also present with centric implantation (91) and do not reflect the implantation events of higher-order primates. Moreover, while guinea pig embryos invade deeply within the endometrial stroma, there is no post-ovulatory rise in progesterone levels and decidualisation of endometrial stromal cells is induced, rather than spontaneous; characteristics unique to true menstruating species (20). Together, these studies highlight the significant species variations in implantation in the mammals.

While old-world monkeys clearly present with aggressive trophoblast invasion, great-ape embryos invade even deeper within the endometrial stroma. Recently, trophoblast invasion and subsequent spiral artery remodelling in chimpanzees (92) and gorillas (93) were simultaneously described, both strongly resembling the processes in later stages of human implantation. Considering our phylogenetic relatedness, it is perhaps no surprise that deep trophoblast invasion extending to the inner myometrium are features shared between all great apes, and implantation is most suitably modelled in these species.

Clearly, *A. cahirinus* presents with a unique combination of rodent- and human-like reproductive characteristics. It is the only menstruating rodent that also exhibits postpartum ovulation, an absence of lactational amenorrhea and shallow implantation, and yet it clearly shows remodelling of spiral arteries during pregnancy (87); these are perhaps the most puzzling combinations of reproductive traits observed to date. This not only questions the dogma of the origins of menstruation in mammalian species, but also challenges the assumption that menstruation evolved alongside aggressive trophoblast invasion. It remains to be determined the depth of invasion and role of early formation of the placenta in this species. These observations will be vital in establishing this species as a relevant and accessible animal model of female reproductive health.

## Assisted Reproductive Technologies: a Need For Female Focus

Infertility is an incredibly heterogenous, multifaceted condition. Today, it is estimated that 8–12% of couples of reproductive age experience infertility and require various interventions and technologies to conceive (94). The use of Assisted Reproductive Technologies (ARTs) have been used extensively to treat failures in human reproduction since their rapid development, refinement, and accessibility over the past half century. ARTs involve the manipulation of male and female gametes to achieve a viable pregnancy and, ultimately, a live birth. The most commonly used of these reproductive techniques include *in vitro fertilisation* (IVF) and intracytoplasmic sperm injection (ICSI), caused by a growing population of subfertile individuals. Despite growing popularity and treatments becoming more affordable, the success rate, as determined by live birth rate, of ARTs plateaued at around 30% for over the previous decade, and is now declining (95). This is likely to stem from a fundamental knowledge gap in the multiple steps of human fertilisation and implantation, and is further complicated by an inability to conduct invasive procedures and long-term research, particularly in women.

Gamete collection is at the apex of many ART procedures. While semen is relatively easy to collect and study, collection of mature oocytes suitable for *in-vitro* research is far more difficult, and much of our understanding of female-factor infertility has relied on comparative research in farm and laboratory species. Collecting healthy, mature oocytes is a critical step for *in-vitro* research, animal colony management, and treatment of infertility. However, in most cases, a low natural ovulation rate is inefficient and has led to the development of controlled ovarian stimulation (COS) or superovulation. This is turn has led to a significant increased incidence in COS-related complications such as ovarian hyperstimulation syndrome (OHSS) (96). Further research is therefore required to mitigate or prevent the potentially lethal outcomes of COS. While clinical studies are possible, the ability to screen for these complications and develop novel treatment regimens in an appropriate animal model would not only increase likelihood of success but reduce physical and emotional discomfort for infertility patients.

Another hurdle in understanding human infertility is the fertilisation of mature oocytes *in vitro* (IVF) and *in vivo*. While human IVF is a broadly successful technique, fertilisation rates in humans rarely exceed 70% (97, 98), and some patients, although rare, still present with total fertilisation failure (TFF) (99). Deciphering the aetiology of TFF remains technically challenging; particularly as progress in human IVF is hindered by ethical and legal restrictions limiting access to human tissues. IVF in farm and laboratory species has provided substantial evidence for male- and female-factors affecting fertilisation success including DNA fragmentation, inadequate sperm capacitation, aneuploidy or poor oocyte activation (100). In some patients, however, ICSI is sought as the course of action, even if evidence of male-factor fertility is lacking, in a bid to overcome cases of idiopathic infertility (101). While ICSI is an effective treatment for severe male-factor infertility, ICSI does not confer any benefit in cycles with female factor infertility (97, 98, 101), which ignores half of the contributing parameters for fertility failure.

Of non-primate models, bovine, murine and cricetinae oocytes are typically obtained for *in vitro* studies, with rodents often preferred for their ready availability in most research settings. However, compared to the primate, cow or even hamster oocyte, mouse oocytes have a poor “wound healing” capacity following mechanical manipulation linked to ICSI, and survival rarely exceeds 50% (102, 103). Similarly, ICSI in cows is very rarely performed due to the technical complexity, and additional requirement of oocyte activation following manipulation (104). Although ICSI is successful in non-human primates (105–107), the combined cost of maintaining captive primate colonies and the complexity of ICSI limits their use in biomedical research. Clearly, current animal models of ICSI and fertilisation failure present several technical and biological issues that limit their application as models for clinical use. The spiny mouse has demonstrated the ability to wound heal regions of damaged skin and hair follicles completely scar-free (32). The potential relationship between this and the similar scar-free healing of the endometrium during the menstrual cycle has been contemplated elsewhere (28), but certainly warrants further exploration. Whether or not these wound healing capabilities apply to the oocytes of the spiny mouse remains to be seen.

Most of the current library of ART knowledge has been derived from non-menstruating animal models. The history of assisted reproduction is now more than 120 years old (108). Much of the early, ground-breaking work on *in-vitro* fertilisation (IVF) and embryo culture were performed in rodents and cattle (100, 109). The obvious relevance issue for these models is that they exhibit an oestrous, not a menstrual, cycle, and that they do not experience a lifetime of cyclical endometrial shedding and regeneration that may have unforeseen implications for the ability of a couple to conceive and carry a baby to term. An influx of inflammatory cytokines, proteases, repair molecules triggered by cascading hormones (110) are all important in the preparation of the uterus for implantation, but paired with foreign invading spermatozoa and lifestyle factors, the ability to conceive varies greatly within our own species. Even great apes, our closest evolutionary relatives, present copious challenges, largely due to the complexities of their husbandry and welfare needs, high running costs for their maintenance and logistical challenges in combating societal and ethical concerns to justify their usage (100).

The spiny mouse provides many novel features that are important and useful in providing an alternate model for ARTs research. Their small size and relatively low cost combined with similarities to human uterine physiology in terms of cyclical endometrial differentiation provides a relevant model to comprehensively investigate currently ambiguous clinical practises. One such practise is the use of endometrial scratch in ART, where the uterine lining is subjected to physical injury prior to or during an ART cycle. The initial observation of improved pregnancy and live birth rates led to a rapid adoption of the procedure (111, 112). Lensen et al. determining 83% of clinics in the UK, Australia and New Zealand recommend this to ART patients (113), despite conflicting evidence of its efficacy in multiple randomised controlled trials (RCTs). In fact, a recent systematic review comparing women undergoing endometrial scratching with no prior IVF/ICSI treatment, one or two failed cycles found no significant differences between scratching and controls (114). The authors advocate at best, endometrial scratching provides false hope for patients and with such poor evidence, could in fact be harmful. They also conclude that the heterogeneity of patients in the analysed RCTs is a major limitation. Furthermore, the behaviour of the endometrium in terms of vascular and stromal inflammation and repair in response to the scratch would likely be associated with that seen during menstruation; thus, subjective to the immune and hormonal profile of each individual [see (11)]. Here is where the spiny mouse as a preclinical model presents obvious advantages for ART procedure development and refinement.

## Conclusion

The discovery of menstruation in the spiny mouse offers an exciting opportunity to advance studies in many areas of female reproductive biology. We now have access to a more physiologically relevant, small animal model for menstrual research. In a broader sense, it also provides us with a unique opportunity to develop new disease models for AUB, infertility and preeclampsia and to provide a new and, in some areas of research, better model to test and develop new ideas to improve our understanding of other difficult issues in women's health.

## Author Contributions

NB developed theories and drafted manuscript. JM contributed to manuscript subsections, figures, and editing. SE and PT-S refined ideas and edited manuscript. All authors contributed to the article and approved the submitted version.

## Funding

Hudson Institute of Medical Research was supported by Victorian Government Operational Infrastructure funding.

## Conflict of Interest

The authors declare that the research was conducted in the absence of any commercial or financial relationships that could be construed as a potential conflict of interest.

## Publisher's Note

All claims expressed in this article are solely those of the authors and do not necessarily represent those of their affiliated organizations, or those of the publisher, the editors and the reviewers. Any product that may be evaluated in this article, or claim that may be made by its manufacturer, is not guaranteed or endorsed by the publisher.

## References

[1] Hamlett G. Uterine bleeding in a bat, *Glossophaga soricina*. Anat Rec. (1934) 60:9–17. 10.1002/ar.109060010425855820

[2] Van der Horst C Gillman J. The menstrual cycle in Elephantulus. S Afr J Med Sci. (1941) 6:27–42.

[3] Rasweiler JJ. Spontaneous decidual reactions and menstruation in the black mastiff bat, Molossus ater. Am J Anat. (1991) 191:1–22. 10.1002/aja.1001910102 2063806

[4] Rasweiler JJ De Bonilla H. Menstruation in short-tailed fruit bats (*Carollia* spp.). J Reprod Fertil. (1992) 95:231–48. 10.1530/jrf.0.0950231 1625240

[5] Zhang X Zhu C Lin H Yang Q Ou Q Li Y. Wild fulvous fruit bats (*Rousettus leschenaulti*) exhibit human-like menstrual cycle. Biol Reprod. (2007) 77:358–64. 10.1095/biolreprod.106.058958 17494915

[6] Carson DD Bagchi I Dey SK Enders AC Fazleabas AT Lessey BA. Embryo implantation. Dev Biol. (2000) 223:217–37. 10.1006/dbio.2000.9767 10882512

[7] Dufour DL Sauther ML. Comparative and evolutionary dimensions of the energetics of human pregnancy and lactation. Am J Hum Biol. (2002) 14:584–602. 10.1002/ajhb.10071 12203813

[8] Ulijaszek SJ. Comparative energetics of primate fetal growth. Ame J Hum Biol. (2002) 14:603–8. 10.1002/ajhb.10088 12203814

[9] Carter AM Enders AC Pijnenborg R. The role of invasive trophoblast in implantation and placentation of primates. Philos Transac R Soc B Biol Sci. (2015) 370:20140070. 10.1098/rstb.2014.0070 25602074 PMC4305171

[10] Bellofiore N Ellery SJ Mamrot J Walker DW Temple-Smith P Dickinson H. First evidence of a menstruating rodent: the spiny mouse (*Acomys cahirinus*). Am J Obstetr Gynecol. (2017) 216:40. e41-11. 10.1016/j.ajog.2016.07.041 27503621

[11] Bellofiore N Rana S Dickinson H Temple-Smith P Evans J. Characterization of human-like menstruation in the spiny mouse: comparative studies with the human and induced mouse model. Hum Reprod. (2018) 33:1715–26. 10.1093/humrep/dey247 30032205

[12] Bellofiore N Cousins F Temple-Smith P Evans J. Altered exploratory behaviour and increased food intake in the spiny mouse before menstruation: a unique pre-clinical model for examining premenstrual syndrome. Hum Reprod. (2019) 34:308–22. 10.1093/humrep/dey360 30561655

[13] O'Connell BA Moritz KM Walker DW Dickinson H. Sexually dimorphic placental development throughout gestation in the spiny mouse (*Acomys cahirinus*). Placenta. (2013) 34:119–26. 10.1016/j.placenta.2012.11.009 23260227

[14] Ellery SJ LaRosa DA Kett MM Della Gatta PA Snow RJ Walker DW. Maternal creatine homeostasis is altered during gestation in the spiny mouse: is this a metabolic adaptation to pregnancy? BMC Pregn Childbirth. (2015) 15:1. 10.1186/s12884-015-0524-1 25885219 PMC4423481

[15] Murray CJ Lopez AD. Global health statistics: a compendium of incidence, prevalence and mortality estimates for over 200 conditions. In: Harvard School of Public Health on behalf of the World Health Organization and the World Bank, editor. Global Health Statistics: A Compendiumof Incidence, Prevalence and Mortality Estimates for Over 200 Conditions. Boston (1996). p. 1–906.

[16] Murray CJ Lopez AD World Health Organization. The Global Burden of Disease: A Comprehensive Assessment of Mortality and Disability From Diseases, Injuries, and Risk Factors in 1990 and Projected to 2020: Summary. World Health Organization (1996).

[17] Munro MG Critchley HO Broder MS Fraser IS; FIGO Working Group on Menstrual Disorders. FIGO classification system (PALM-COEIN) for causes of abnormal uterine bleeding in nongravid women of reproductive age. Int J Gynecol Obstetr. (2011) 113:3–13. 10.1016/j.ijgo.2010.11.011 21345435

[18] Liu Z Doan QV Blumenthal P Dubois RW. A systematic review evaluating health-related quality of life, work impairment, and health-care costs and utilization in abnormal uterine bleeding. Value Health. (2007) 10:183–94. 10.1111/j.1524-4733.2007.00168.x 17532811

[19] Johnson MH. Essential Reproduction. Hoboken, NJ: JohnWiley and Sons (2018).

[20] Bellofiore N Cousins F Temple-Smith P Dickinson H Evans J. A missing piece: the spiny mouse and the puzzle of menstruating species. J Mol Endocrinol. (2018) 61:R25–41. 10.1530/JME-17-0278 29789322

[21] Salker M Teklenburg G Molokhia M Lavery S Trew G Aojanepong T. Natural selection of human embryos: impaired decidualization of endometrium disables embryo-maternal interactions and causes recurrent pregnancy loss. PLoS ONE. (2010) 5:e10287. 10.1371/journal.pone.0010287 20422017 PMC2858209

[22] Teklenburg G Salker M Molokhia M Lavery S Trew G Aojanepong T. Natural selection of human embryos: decidualizing endometrial stromal cells serve as sensors of embryo quality upon implantation. PLoS ONE. (2010) 5:e10258. 10.1371/journal.pone.0010258 20422011 PMC2858159

[23] Emera D Romero R Wagner G. The evolution of menstruation: a new model for genetic assimilation. Bioessays. (2012) 34:26–35. 10.1002/bies.201100099 22057551 PMC3528014

[24] Gellerson B Brosens JJ. Cyclic decidualisation of the human endometrium in reproductive health and failure. Endocr Rev. (2014) 35:851–905. 10.1210/er.2014-1045 25141152

[25] Finn C. Menstruation: a nonadaptive consequence of uterine evolution. Q Rev Biol. (1998) 73:163–73. 10.1086/420183 9618925

[26] Demir R Yaba A Huppertz B. Vasculogenesis and angiogenesis in the endometrium during menstrual cycle and implantation. Acta Histochem. (2010) 112:203–14. 10.1016/j.acthis.2009.04.004 19481785

[27] Van Der Horst C Gillman J. The menstrual cycle in Elephantulus. South Afr J Med Sci. (1941) 6:27–42.

[28] Bellofiore N Evans J. Monkeys, mice and menses: the bloody anomaly of the spiny mouse. J Assist Reprod Genet. (2019) 36:811–17. 10.1007/s10815-018-1390-3 30610663 PMC6541669

[29] Haughton CL Gawriluk TR Seifert AW. The biology and husbandry of the African spiny mouse (*Acomys cahirinus*) and the research uses of a laboratory colony. J Am Assoc Lab Anim Sci. (2016) 55:9–17. 26817973 PMC4747004

[30] Gonet AE Stauffacher W Pictet R Renold AE. Obesity and diabetes mellitus with striking congenital hyperplasia of the islets of Langerhans in spiny mice (*Acomys cahirinus*). Diabetologia. (1966) 1:162–71. 10.1007/BF01257907 24173297

[31] Strasser H. A breeding program for spontaneously diabetic experimental animals: *Psammomys obesus* (sand rat) and *Acomys cahirinus* (spiny mouse). Lab Anim Care. (1968) 18:328. 4233365

[32] Seifert AW Kiama SG Seifert MG Goheen JR Palmer TM Maden M. Skin shedding and tissue regeneration in African spiny mice (Acomys). Nature. (2012) 489:561–5. 10.1038/nature11499 23018966 PMC3480082

[33] Dickinson H Walker D. Managing a colony of spiny mice (*Acomys cahirinus*) for perinatal research. Austral N Zeal Council Care Anim Res Train News. (2007) 20:4–11.

[34] Lamers WH Mooren PG Griep H Endert E Degenhart HJ Charles R. Hormones in perinatal rat and spiny mouse: relation to altricial and precocial timing of birth. Am J Physiol Endocrinol Metab. (1986) 251:E78–85. 10.1152/ajpendo.1986.251.1.E78 3524260

[35] Quinn TA Ratnayake U Dickinson H Nguyen TH McIntosh M Castillo-Melendez M. Ontogeny of the adrenal gland in the spiny mouse, with particular reference to production of the steroids cortisol and dehydroepiandrosterone. Endocrinology. (2013) 154:1190–201. 10.1210/en.2012-1953 23354096

[36] Oosterhuis W Mooren P Charles R Lamers W. Perinatal development of the lung in rat and spiny mouse: its relation to altricial and precocial timing of birth. Neonatology. (1984) 45:236–43. 10.1159/000242011 6326867

[37] Hułas M Gawron A Orfin G. A comparative study of ovary development in the precocial spiny mouse (*Acomys cahirinus*) and the altricial Norway rat (*Rattus norvegicus*). Israel J Ecol Evol. (2003) 49:307–13. 10.1560/GUDG-6MCP-RETL-7FA5

[38] Dickinson H Walker DW Cullen-McEwen L Wintour EM Moritz K. The spiny mouse (*Acomys cahirinus*) completes nephrogenesis before birth. Am J Physiol Renal Physiol. (2005) 289:F273–9. 10.1152/ajprenal.00400.2004 15741606

[39] Ellery SJ LaRosa DA Kett MM Della Gatta PA Snow RJ Walker DW. Dietary creatine supplementation during pregnancy: a study on the effects of creatine supplementation on creatine homeostasis and renal excretory function in spiny mice. Amino Acids. (2016) 48:1819–30. 10.1007/s00726-015-2150-7 26695944

[40] Dickinson H Ellery S Davies-Tuck M Tolcos M Nitsos I Walker D. Description of a method for inducing fetal growth restriction in the spiny mouse. J Dev Orig Health Dis. (2017) 8:550–5. 10.1017/S2040174417000393 28659226

[41] Finn C Pope M. Vascular and cellular changes in the decidualized endometrium of the ovariectomized mouse following cessation of hormone treatment: a possible model for menstruation. J Endocrinol. (1984) 100:295. 10.1677/joe.0.1000295 6699534

[42] Brasted M White C Kennedy T Salamonsen L. Mimicking the events of menstruation in the murine uterus. Biol Reprod. (2003) 69:1273–80. 10.1095/biolreprod.103.016550 12801986

[43] Rudolph M Döcke W-D Müller A Menning A Röse L Zollner TM. Induction of overt menstruation in intact mice. PLoS ONE. (2012) 7:e32922. 10.1371/journal.pone.0032922 22412950 PMC3296749

[44] Wang Q Xu X He B Li Y Chen X Wang J. A critical period of progesterone withdrawal precedes endometrial breakdown and shedding in mouse menstrual-like model. Hum Reprod. (2013) 28:1670–8. 10.1093/humrep/det052 23512993

[45] Greaves E Cousins FL Murray A Esnal-Zufiaurre A Fassbender A Horne AW. A novel mouse model of endometriosis mimics human phenotype and reveals insights into the inflammatory contribution of shed endometrium. Am J Pathol. (2014) 184:1930–9. 10.1016/j.ajpath.2014.03.011 24910298 PMC4076466

[46] Cousins FL Kirkwood PM Murray AA Collins F Gibson DA Saunders PT. Androgens regulate scarless repair of the endometrial “wound” in a mouse model of menstruation. FASEB J. (2016) 30:2802–11. 10.1096/fj.201600078R 27121597

[47] Lin Y-J Lai M-D Lei H-Y Wing L-YC. Neutrophils and macrophages promote angiogenesis in the early stage of endometriosis in a mouse model. Endocrinology. (2006) 147:1278–86. 10.1210/en.2005-0790 16306083

[48] Pelch KE Schroder AL Kimball PA Sharpe-Timms KL Davis JW Nagel SC. Aberrant gene expression profile in a mouse model of endometriosis mirrors that observed in women. Fertil Steril. (2010) 93:1615–27.e1618. 10.1016/j.fertnstert.2009.03.086 19473656 PMC2904074

[49] Higham JM O'brien P Shaw R. Assessment of menstrual blood loss using a pictorial chart. BJOG Int J Obstetr Gynaecol. (1990) 97:734–9. 10.1111/j.1471-0528.1990.tb16249.x 2400752

[50] Abbott D Bird I. Nonhuman Primates as models for human adrenal androgen production: function and dysfunction. Rev Endocr Metab Disord. (2009) 10:33–42. 10.1007/s11154-008-9099-8 18683055 PMC2653599

[51] Nakamura Y Gang HX Suzuki T Sasano H Rainey WE. Adrenal changes associated with adrenarche. Rev Endocr Metab Disord. (2009) 10:19–26. 10.1007/s11154-008-9092-2 18821019 PMC3712864

[52] Conley AJ Pattison JC Bird IM. Variations in adrenal androgen production among (nonhuman) primates. Semin Reprod Med. (2004) 22:311–26. 10.1055/s-2004-861548 15635499

[53] Pattison JC. Marmoset 17 [Alpha]-Hydoxylase/17, 20-Lyase Cytochrome P450: Relationship Between Enzyme Structure and Function to Low Circulating DHEA Levels Observed in vivo. Madison, WI: University of Wisconsin (2008).

[54] Gibson DA Simitsidellis I Kelepouri O Critchley HO Saunders PT. Dehydroepiandrosterone enhances decidualization in women of advanced reproductive age. Fertil Steril. (2018) 109:728–34. e722. 10.1016/j.fertnstert.2017.12.024 29397924 PMC5908781

[55] Labrie F Labrie C. DHEA and intracrinology at menopause, a positive choice for evolution of the human species. Climacteric. (2013) 16:205–13. 10.3109/13697137.2012.733983 23126249

[56] Miller KKM Al-Rayyan N Ivanova MM Mattingly KA Ripp SL Klinge CM. DHEA metabolites activate estrogen receptors alpha and beta. Steroids. (2013) 78:15–25. 10.1016/j.steroids.2012.10.002 23123738 PMC3529809

[57] Mills SJ Ashworth JJ Gilliver SC Hardman MJ Ashcroft GS. The sex steroid precursor DHEA accelerates cutaneous wound healing via the estrogen receptors. J Investig Dermatol. (2005) 125:1053–62. 10.1111/j.0022-202X.2005.23926.x 16297209

[58] Quinn TA Ratnayake U Dickinson H Castillo-Melendez M Walker DW. The feto-placental unit, and potential roles of dehydroepiandrosterone (DHEA) in prenatal and postnatal brain development: a re-examination using the spiny mouse. J Steroid Biochem Mol Biol. (2016) 160:204–13. 10.1016/j.jsbmb.2015.09.044 26485665

[59] Solerte SB Ferrari E Cuzzoni G Locatelli E Giustina A Zamboni M. Decreased release of the angiogenic peptide vascular endothelial growth factor in Alzheimer's disease: recovering effect with insulin and DHEA sulfate. Dement Geriatr Cogn Disord. (2005) 19:1–10. 10.1159/000080963 15383738

[60] Liu D Iruthayanathan M Homan LL Wang Y Yang L Wang Y. Dehydroepiandrosterone stimulates endothelial proliferation and angiogenesis through extracellular signal-regulated kinase 1/2-mediated mechanisms. Endocrinology. (2008) 149:889–98. 10.1210/en.2007-1125 18079198 PMC2275364

[61] Walters K McTavish KJ Seneviratne M Jimenez M McMahon A Allan C. Subfertile female androgen receptor knockout mice exhibit defects in neuroendocrine signaling, intraovarian function, and uterine development but not uterine function. Endocrinology. (2009) 150:3274–82. 10.1210/en.2008-1750 19359383 PMC2703552

[62] Wang L Wang Y-D Wang W-J Li D-J. Differential regulation of dehydroepiandrosterone and estrogen on bone and uterus in ovariectomized mice. Osteoporosis Int. (2009) 20:79–92. 10.1007/s00198-008-0631-1 18690485

[63] Abalos E Cuesta C Grosso AL Chou D Say L. Global and regional estimates of preeclampsia and eclampsia: a systematic review. Eur J Obstet Gynecol Reprod Biol. (2013) 170:1–7. 10.1016/j.ejogrb.2013.05.005 23746796

[64] Young BC Levine RJ Karumanchi SA. Pathogenesis of preeclampsia. Ann Rev Pathol Mech Dis. (2010) 5:173–92. 10.1146/annurev-pathol-121808-102149 20078220

[65] Backes CH Markham K Moorehead P Cordero L Nankervis CA Giannone PJ. (2011). Maternal preeclampsia and neonatal outcomes. J Pregn. (2011). 10.1155/2011/214365 21547086 PMC3087144

[66] Ghulmiyyah L Sibai B. Maternal mortality from preeclampsia/eclampsia. Semin Perinatol. (2012) 36:56–9. 10.1053/j.semperi.2011.09.011 22280867

[67] Platt M. Outcomes in preterm infants. Public Health. (2014) 128:399–403. 10.1016/j.puhe.2014.03.010 24794180

[68] Pijnenborg R Vercruysse L Hanssens M. The uterine spiral arteries in human pregnancy: facts and controversies. Placenta. (2006) 27:939–58. 10.1016/j.placenta.2005.12.006 16490251

[69] Huppertz B. Maternal and fetal factors and placentation: implications for pre-eclampsia. Pregn Hypertens Int J Women's Cardiovasc Health. (2014) 4:244. 10.1016/j.preghy.2014.04.015 26104643

[70] Dekker GA Sibai BM. Etiology and pathogenesis of preeclampsia: current concepts. Am J Obstet Gynecol. (1998) 179:1359–75. 10.1016/S0002-9378(98)70160-7 9822529

[71] Kaufmann P Black S Huppertz B. Endovascular trophoblast invasion: implications for the pathogenesis of intrauterine growth retardation and preeclampsia. Biol Reprod. (2003) 69:1–7. 10.1095/biolreprod.102.014977 12620937

[72] Zhou Y Damsky CH Fisher SJ. Preeclampsia is associated with failure of human cytotrophoblasts to mimic a vascular adhesion phenotype. One cause of defective endovascular invasion in this syndrome? J Clin Invest. (1997) 99:2152–64. 10.1172/JCI119388 9151787 PMC508045

[73] Lyall F Greer I Boswell F Young A Macara L Jeffers M. Expression of cell adhesion molecules in placentae from pregnancies complicated by pre-eclampsia and intrauterine growth retardation. Placenta. (1995) 16:579–87. 10.1016/0143-4004(95)90027-6 8577657

[74] Chwalisz K Ciesla I Garfield R. Inhibition of nitric oxide (NO) synthesis induces preterm parturition and preeclampsia-like conditions in guinea pigs. In: Program of the 41st Meeting of the Society of Gynecologic Investigation. Chicago, IL (1994).

[75] Garfield R Yallampalli C Buhimschi I Chwalisz K. Reversal of preeclampsia symptoms induced in rats by nitric oxide inhibition with L-arginine, steroid hormones and an endothelin antagonist. In: Program of the 41st Meeting of the Society of Gynecologic Investigation. Chicago, IL (1994). p. 384.

[76] Nanaev A Chwalisz K Frank H-G Kohnen G Hegele-Hartung C Kaufmann P. Physiological dilation of uteroplacental arteries in the guinea pig depends on nitric oxide synthase activity of extravillous trophoblast. Cell Tissue Res. (1995) 282:407–21. 10.1007/BF00318873 8581935

[77] Brosens JJ Parker MG McIndoe A Pijnenborg R Brosens IA. A role for menstruation in preconditioning the uterus for successful pregnancy. Am J Obstetr Gynecol. (2009) 615.e611–16. 10.1016/j.ajog.2008.11.037 19136085

[78] Harmon AC Cornelius DC Amaral LM Faulkner JL Cunningham MW Jr. Wallace K. The role of inflammation in the pathology of preeclampsia. Clin Sci. (2016) 130:409–19. 10.1042/CS20150702 26846579 PMC5484393

[79] Dal J Vural B Caliskan E Ozkan S Yucesoy I. Power Doppler ultrasound studies of ovarian, uterine, and endometrial blood flow in regularly menstruating women with respect to luteal phase defects. Fertil Steril. (2005) 84:224–7. 10.1016/j.fertnstert.2004.12.059 16009189

[80] Schatz F Guzeloglu-Kayisli O Arlier S Kayisli UA Lockwood CJ. The role of decidual cells in uterine hemostasis, menstruation, inflammation, adverse pregnancy outcomes and abnormal uterine bleeding. Hum Reprod Update. (2016) 22:497–515. 10.1093/humupd/dmw004 26912000 PMC4917742

[81] Brosens I Muter J Ewington L Puttemans P Petraglia F Brosens JJ. Adolescent preeclampsia: pathological drivers and clinical prevention. Reprod Sci. (2019) 26:159–71. 10.1177/1933719118804412 30317927

[82] Erez O Vardi IS Hallak M Hershkovitz R Dukler D Mazor M. Preeclampsia in twin gestations: association with IVF treatments, parity and maternal age. J Maternal Fetal Neonatal Med. (2006) 19:141–6. 10.1080/14767050500246045 16690506

[83] Bdolah Y Lam C Rajakumar A Shivalingappa V Mutter W Sachs BP. Twin pregnancy and the risk of preeclampsia: bigger placenta or relative ischemia? Am J Obstetr Gynecol. (2008) 198:428.e421-6. 10.1016/j.ajog.2007.10.783 18191808

[84] Chen X-K Wen SW Bottomley J Smith GN Leader A Walker MC. *In vitro* fertilization is associated with an increased risk for preeclampsia. Hypertens Pregn. (2009) 28:1–12. 10.1080/10641950802001859 19165665

[85] Hernández-Díaz S Toh S Cnattingius S. Risk of pre-eclampsia in first and subsequent pregnancies: prospective cohort study. BMJ. (2009) 338:b2255. 10.1136/bmj.b2255 19541696 PMC3269902

[86] Marshall SA Hannan NJ Jelinic M Nguyen TP Girling JE Parry LJ. Animal models of preeclampsia: translational failings and why. Am J Physiol Regul Integr Comp Physiol. (2018) 314:R499–508. 10.1152/ajpregu.00355.2017 29212809

[87] McKenna J Bellofiore N Dimitriadis E Temple-Smith P. Postpartum ovulation and early pregnancy in the menstruating spiny mouse, Acomys cahirinus. Sci Rep. (2021) 11:1–11. 10.1038/s41598-021-84361-z 33674629 PMC7935856

[88] Nimbkar-Joshi S Rosario G Katkam R Manjramkar D Metkari S Puri CP. Embryo-induced alterations in the molecular phenotype of primate endometrium. J Reprod Immunol. (2009) 83:65–71. 10.1016/j.jri.2009.08.011 19880195

[89] Lee KY DeMayo FJ. Animal models of implantation. Reproduction. (2004) 128:679–95. 10.1530/rep.1.00340 15579585

[90] Franek A Salamonsen L Lopata A. Marmoset monkey trophoblastic tissue growth and matrix metalloproteinase secretion in culture. Reproduction. (1999) 117:107–14. 10.1530/jrf.0.1170107 10645251

[91] Enders AC Lopata A. Implantation in the marmoset monkey: expansion of the early implantation site. Anat Rec. (1999) 256:279–99. 10521786 10.1002/(SICI)1097-0185(19991101)256:3<279::AID-AR7>3.0.CO;2-O

[92] Pijnenborg R Vercruysse L Carter AM. Deep trophoblast invasion and spiral artery remodelling in the placental bed of the chimpanzee. Placenta. (2011) 32:400–8. 10.1016/j.placenta.2011.02.009 21459441

[93] Pijnenborg R Vercruysse L Carter AM. Deep trophoblast invasion and spiral artery remodelling in the placental bed of the lowland gorilla. Placenta. (2011) 32:586–91. 10.1016/j.placenta.2011.05.007 21705078

[94] Veiga A Gianaroli L Ory S Horton M Feinberg E Penzias A. Assisted reproduction and COVID-19: A joint statement of ASRM, ESHRE and IFFS*. Fertil Steril. (2020) 114:484–5. 10.1016/j.fertnstert.2020.06.044 32674808 PMC7355315

[95] Gleicher N Kushnir VA Barad DH. Worldwide decline of IVF birth rates and its probable causes. Hum Reprod Open. (2019) 2019:hoz017. 10.1093/hropen/hoz017 31406934 PMC6686986

[96] Aboulghar M. Prediction of ovarian hyperstimulation syndrome (OHSS) Estradiol level has an important role in the prediction of OHSS. Hum Reprod. (2003) 18:1140–1. 10.1093/humrep/deg208 12773437

[97] Bhattacharya S Hamilton M Shaaban M Khalaf Y Seddler M Ghobara T. Conventional *in-vitro* fertilisation versus intracytoplasmic sperm injection for the treatment of non-male-factor infertility: a randomised controlled trial. Lancet. (2001) 357:2075–9. 10.1016/S0140-6736(00)05179-5 11445099

[98] De Munck N El Khatib I Abdala A El-Damen A Bayram A Arnanz A. Intracytoplasmic sperm injection is not superior to conventional IVF in couples with non-male factor infertility and preimplantation genetic testing for aneuploidies (PGT-A). Hum Reprod. (2020) 35:317–27. 10.1093/humrep/deaa002 32086522

[99] Suo L Xiao Zhou Y Ling Jia L Bo Wu H Zheng J Feng Lyu Q. Transcriptome profiling of human oocytes experiencing recurrent total fertilization failure. Sci Rep. (2018) 8:1–11. 10.1038/s41598-018-36275-6 30559372 PMC6297154

[100] Schatten H Constantinescu GM. AnimalModels and Human Reproduction. Hoboken, NJ: John Wiley and Sons (2017).

[101] Supramaniam P Granne I Ohuma E Lim L McVeigh E Venkatakrishnan R. ICSI does not improve reproductive outcomes in autologous ovarian response cycles with non-male factor subfertility. Hum Reprod. (2020) 35:583–94. 10.1093/humrep/dez301 32161952

[102] Kimura Y Yanagimachi R. Intracytoplasmic sperm injection in the mouse. Biol Reprod. (1995) 52:709–20. 10.1095/biolreprod52.4.709 7779992

[103] Yoshida N Perry AC. Piezo-actuated mouse intracytoplasmic sperm injection (ICSI). Nat Protoc. (2007) 2:296–304. 10.1038/nprot.2007.7 17406589

[104] Emuta C Horiuchi T. Effects of timing of activation and aging of bovine oocytes fertilized by intracytoplasmic sperm injection (ICSI) on cleavage and subsequent embryonic development *in vitro*. J Reprod Dev. (2001) 47:399–405. 10.1262/jrd.47.399

[105] Wolf D Thormahlen S Ramsey C Yeoman R Fanton J Mitalipov S. Use of assisted reproductive technologies in the propagation of rhesus macaque offspring. Biol Reprod. (2004) 71:486–93. 10.1095/biolreprod.103.025932 15044263

[106] Sun Q Dong J Yang W Jin Y Yang M Wang Y. Efficient reproduction of cynomolgus monkey using pronuclear embryo transfer technique. Proc Nat Acad Sci. (2008) 105:12956–60. 10.1073/pnas.0805639105 18725640 PMC2529107

[107] Simerly CR Castro CA Jacoby E Grund K Turpin J McFarland D. Assisted Reproductive Technologies (ART) with baboons generate live offspring: a nonhuman primate model for ART and reproductive sciences. Reprod Sci. (2010) 17:917–30. 10.1177/1933719110374114 20631291 PMC3307099

[108] Heape W. Preliminary note on the transplantation and growth of mammalian ova within a uterine foster-mother. Proc R Soc Lond. (1890) 48:457–8. 10.1098/rspl.1890.0053

[109] Bavister BD. Culture of preimplantation embryos: facts and artifacts. Hum Reprod Update. (1995) 1:91–148. 10.1093/humupd/1.2.91 15726768

[110] Salamonsen LA. Tissue injury and repair in the female human reproductive tract. Reproduction. (2003) 125:301–11. 10.1530/rep.0.1250301 12611594

[111] Granot I Dekel N Bechor E Segal I Fieldust S Barash A. Temporal analysis of connexin43 protein and gene expression throughout the menstrual cycle in human endometrium. Fertil Steril. (2000) 73:381–6. 10.1016/S0015-0282(99)00531-2 10685547

[112] Barash A Dekel N Fieldust S Segal I Schechtman E Granot I. Local injury to the endometrium doubles the incidence of successful pregnancies in patients undergoing *in vitro* fertilization. Fertil Steril. (2003) 79:1317–22. 10.1016/S0015-0282(03)00345-5 12798877

[113] Lensen S Sadler L Farquhar C. Endometrial scratching for subfertility: everyone's doing it. Hum Reprod. (2016) 31:1241–4. 10.1093/humrep/dew053 27008891

[114] van Hoogenhuijze NE Kasius JC Broekmans FJM Bosteels J Torrance HL. (2019). Endometrial scratching prior to IVF; does it help and for whom? A systematic review and meta-analysis. Hum Reprod Open. (2019) 1:1–18. 10.1093/hropen/hoy025 30895265 PMC6396643

